# Unusual features of the c-ring of F_1_F_O_ ATP synthases

**DOI:** 10.1038/s41598-019-55092-z

**Published:** 2019-12-06

**Authors:** A. V. Vlasov, K. V. Kovalev, S.-H. Marx, E. S. Round, I. Yu. Gushchin, V. A. Polovinkin, N. M. Tsoy, I. S. Okhrimenko, V. I. Borshchevskiy, G. D. Büldt, Yu. L. Ryzhykau, A. V. Rogachev, V. V. Chupin, A. I. Kuklin, N. A. Dencher, V. I. Gordeliy

**Affiliations:** 1 Research Center for Molecular Mechanisms of Aging and Age-Related Diseases, Moscow Institute of Physics and Technology, Dolgoprudny, Russia; 2 Institut de Biologie Structurale Jean-Pierre Ebel, Université Grenoble Alpes–Commissariat à l’Energie Atomique et aux Energies Alternatives–CNRS, Grenoble, France; 3 Institute of Complex Systems (ICS), ICS-6: Structural Biochemistry, Research Centre Jülich, Jülich, Germany; 4 Institute of Crystallography, RWTH Aachen University, Aachen, Germany; 5 Physikalische Biochemie, Fachbereich Chemie, Technische Universität Darmstadt, Alarich-Weiss-Straße 4, D-64287 Darmstadt, Germany; 6 Department of Biochemistry, University of Zurich, Zurich, Switzerland; 7 Joint Institute for Nuclear Research, Dubna, Russia

**Keywords:** Supramolecular assembly, X-ray crystallography

## Abstract

Membrane integral ATP synthases produce adenosine triphosphate, the universal “energy currency” of most organisms. However, important details of proton driven energy conversion are still unknown. We present the first high-resolution structure (2.3 Å) of the *in meso* crystallized c-ring of 14 subunits from spinach chloroplasts. The structure reveals molecular mechanisms of intersubunit contacts in the c_14_-ring, and it shows additional electron densities inside the c-ring which form circles parallel to the membrane plane. Similar densities were found in all known high-resolution structures of c-rings of F_1_F_O_ ATP synthases from archaea and bacteria to eukaryotes. The densities might originate from isoprenoid quinones (such as coenzyme Q in mitochondria and plastoquinone in chloroplasts) that is consistent with differential UV-Vis spectroscopy of the c-ring samples, unusually large distance between polar/apolar interfaces inside the c-ring and universality among different species. Although additional experiments are required to verify this hypothesis, coenzyme Q and its analogues known as electron carriers of bioenergetic chains may be universal cofactors of ATP synthases, stabilizing c-ring and prevent ion leakage through it.

## Introduction

ATP synthases convert energy of H^+^ electrochemical gradient across the membrane into energy of chemical bonds in ATP molecules by coupling of H^+^ transfer and ATP synthesis. ATP synthases are present in inner mitochondria membranes in eukaryotes or in the plasma membrane in archaea or bacteria. In plants, ATP is also synthesized by ATP synthase by the same mechanism, but in two different organelles, mitochondria and chloroplasts^1,2^. ATP synthases in some species use the sodium ion instead of the proton gradient^3–5^.

All ATP synthases consist of two main parts, F_O_ and F_1_ (Fig. S1)^6^. F_1_ represents the water exposed part of the enzyme where the ATP is generated. F_O_ is composed of a membrane integral part and also the peripheral stalk consisting of two b subunits connecting F_O_ and F_1_. Currently it is known that F_1_ and F_O_ have the subunit composition α_3_β_3_γδε (with a hexameric catalytic α_3_β_3_ part) and ab_2_c_(8–17)_, respectively, in archaea, bacteria, and plants. However, mitochondrial F_O_ in addition to ab_2_c_10_ comprises also e, f, g, i/j, k and l subunits with putative regulatory functions^7^.

One of the first remarkable steps was understanding that the ATP synthesis is dependent on conformational changes of α_3_β_3_ induced by rotation of γ subunit^1,2,7^. Then several studies were approaching the major goal - to determine a high resolution structure of a complete enzyme^8,9^. However, only recently, mainly due to cryo-electron microscopy, the goal was partially reached^6,10^. Very recently the EM studies of the *Saccharomyces cerevisiae* mitochondrial ATP synthase revealed the structure of the F_O_ subunit at 3.6 Å resolution. Still only a part of the subunit *d* (a part of a peripheral stalk of yeast mitochondria ATP-synthases) was resolved and the *l* subunit was non-visible in the cryo-EM map^10^. One more advance was the determination of the structure of the complete chloroplast ATP synthase (cF_1_F_O_ complex) from spinach by cryo–EM where all the 26 currently known protein subunits were resolved at a resolution of 2.9 Å (cF_1_) to 3.4 Å (cF_O_)^6^.

A bottle neck of the ATP synthase structural studies still is F_O_. Available data are of lower resolution than in the case of F_1_ and they usually lack the information on some of the subunits. A complementary approach consists of solving the structure of different subunits of F_O_ to high resolution and then combine them with lower resolution data of a complete F_O_. The most advanced are the studies of the c-ring which is a key part of the rotor. The proton flow passing between the ring and *a*-subunit^2,8^ drives rotation of the c-ring. At present there are high resolution structures of mitochondrial and bacteria c-rings solved to 1.9 and 1.8 Å correspondingly. In opposite, there is no high-resolution structure of a plant c-ring. Previous efforts described in^11–13^ resulted only in 3.4, 3.8 and 6 Å resolution structures of chloroplast c-rings from *Spinacia oleracea* and *Pisum savitum*^11–13^. The lack of necessary structural information does not make it possible to understand in detail what is universal and what is different in the construction and mechanisms of the rotor function in different organisms.

We present the first high-resolution structure of the c_14_-ring of cF_1_F_O_ from spinach solved to 2.3 Å resolution. It provides molecular mechanisms of intersubunit contacts that determine the number of c-subunits in the c_14_-ring from spinach chloroplasts. A remarkable feature of the structure is additional electron densities at 5.4 Å distance from each other inside the c-ring, which form circles, oriented parallel to membrane plane. We demonstrate that such characteristic densities are also present in all available high resolution structures of the c-rings^14–19^. We hypothesize that the densities originate from isoprenoid molecules (presumably quinone isoprenoids, such as coenzyme Q in mitochondria, plastoquinone in chloroplasts and menaquinone in bacteria). It is also consistent with differential UV-Vis spectroscopy of the c-ring samples, unusually large distance between polar/apolar interfaces inside the c-ring and universality of the molecule among different organisms. We hypothesize that coenzyme Q and its analogues known as electron carriers of bioenergetics chains may be universal cofactors of ATP synthases and are necessary for stabilization of their F_O_ rotary parts, prevention of ion leakage through the c_14_-ring and protection of the proton uptake/release center of c subunits against reactive oxygen species.

Such densities were neglected (not mentioned) in the previous publications and correspondingly their biological role was not discussed. We believe that our work will trigger additional studies which will help to understand better the roles of the ATP synthases in the cell function.

## Overall Structure

**Figure 1:**
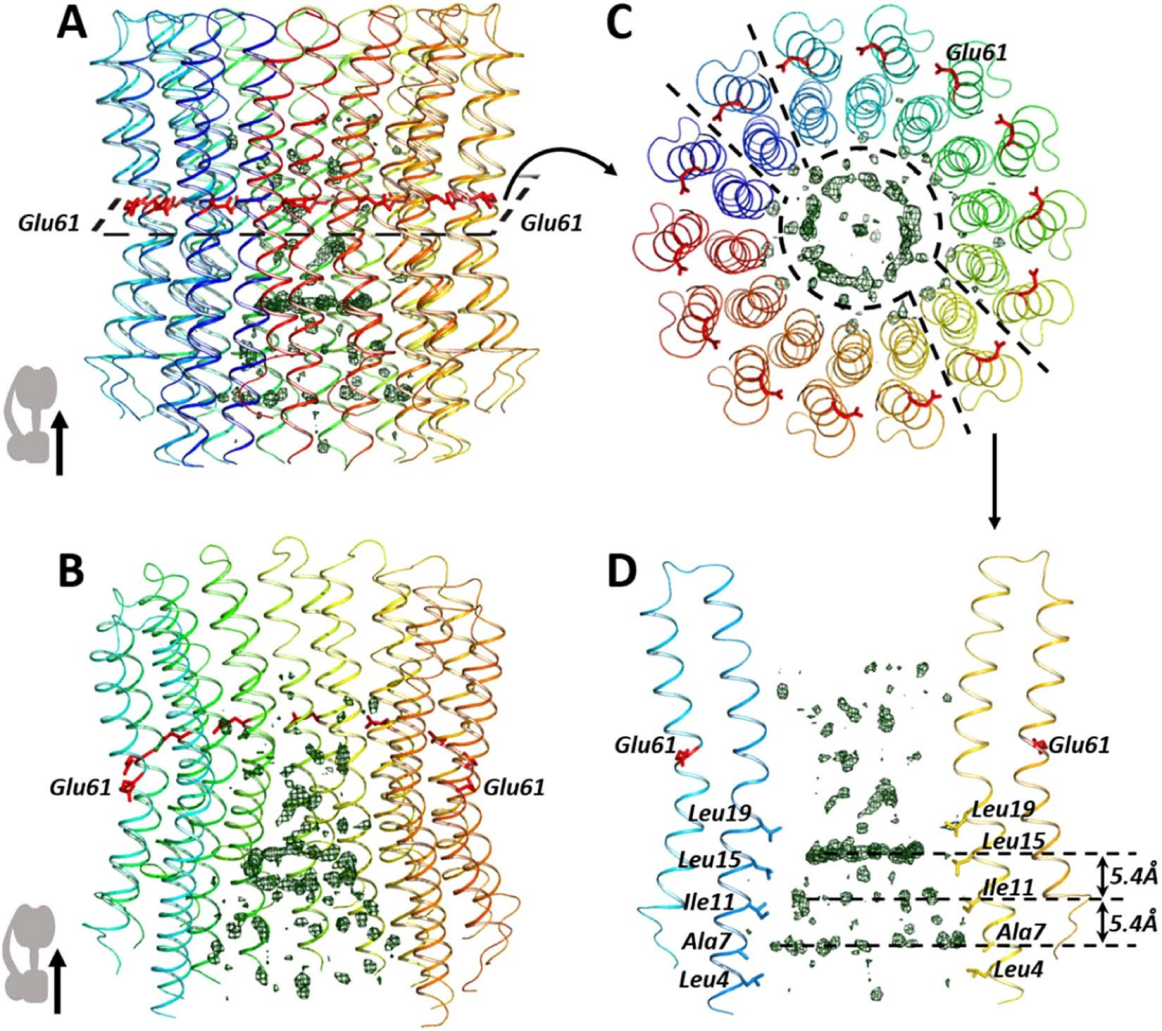
Overall view of the circular positive electron densities inside the chloroplast *c* ring. (**A**) c_14_-ring side view. (**B**) Isometric view of additional electron densities inside the c-ring. (**C**) A clip of the c-ring slightly above Glu61 residues (colored red). (**D**) Section clip shows the additional densities inside the c-ring (deep green mesh density map (Fo – Fc) at 3σ). The distance between each of the three circular densities is 5.4 Å, the same as between amino acids Ala 7, Ile 11, Leu 15 and Leu 19. Stroma of the chloroplast is on the top, thylakoid lumen is on the bottom of the picture, c_1_-subunits are shown as cartoon. The additional densities inside the c-ring are colored as deep green mesh which shows the density map (Fo – Fc) at 3σ.

The complete enzymatically competent cF_1_F_O_ was extracted from spinach, purified and characterized as described in^20,21^. The completeness of the ATP synthase was confirmed by the Blue Native PAGE (Fig. S2A) and used as a starting material for crystallization trials. However, only the c_14_-ring^22,23^ was crystallized upon mixing solubilized cF_1_F_O_ with the monooleoyl based in meso crystallization matrix and using the in meso approach similarly to our previous publications^24–27^. The crystals had a strong yellow color (Fig. S3). Typical for *in meso* crystallization type I crystals (Fig. S4) appeared within 2 months after crystallization setup, and had a plate like shape with the size up to 20 µm. The X-ray data collected with the crystals allowed to determine the structure of the protein at 2.3 Å. The space group of the crystals was determined to be I121, with the cell lattice parameters of a = 93.14 Å, b = 96.34 Å, с = 158.68 Å, α = γ = 90° and β = 106.72°. Data collection and refinement statistics of the spinach chloroplast ATP-synthase c_14_-ring is summarized in Table S1. The c_14_-ring asymmetric unit cell contains one ring with 14 c-subunits in each. The overall structure is shown in Fig. 1A,B.

### Molecular mechanisms of intersubunit contacts in the c-ring

The structure reveals molecular mechanisms of c_1_-c_1_ protomer contacts in the c_14_-ring. It shows a network of hydrogen bonds between water molecules and amino acids showing intersubunit contacts that determine the angle between c_1_ subunits and therefore the number of subunits in the c-ring.

The c_14_-ring has motifs S21xxxG25, G23xxxG27 and G25xxxG29. In the study^28^ the motifs G(A,S)xxxG(A,S) are found to be crucial in case of determination of the c-ring stoichiometry in bacteria and molecular dynamics was used for calculation of hydrogen bonds. Our structure adds details and experimentally shows molecular mechanisms of how this determination occurs in case of c_14_-ring from spinach chloroplasts, in particular, a network of hydrogen bonds between aminoacids and water molecules. Outer helices of the chloroplast c_14_-ring interact only in the region of the active center. Glu61 side chain is directly hydrogen bonded to the Tyr66’ side chain and to the oxygen of Phe59 of the neighbor c-subunit (Fig. S13). In opposite, interactions of inner helices create an extended hydrogen bonding network in the region close to the stroma inside the c_14_-ring, where double conformations of Gln34 side chains of neighbor subunits are connected both directly and via water molecule Wat203. Thr30 side chain and Val26 oxygen are hydrogen bonded via water molecule Wat202, additionally stabilizing the region. A very tight connection occurs between c-subunits close to the hydrophobic region of the inside surface of the c-ring. Ser21 side chain is bonded to the backbone of neighbor c-subunit via water molecule Wat201.

### Positive electron densities inside the c-ring

There are positive electron densities inside the c-ring which form circles parallel to membrane plane (Fig. 1A–D). The additional electron densities form three circles parallel to membrane plane at different levels with the distance of about 5.4 Å between each circle, which corresponds to the average distance between the bulky hydrophobic amino acids of the internal α-helix of the c-subunit (Fig. 1D). These circles of additional electron densities have a coaxial central symmetry and are opposite to the grooves between the bulky hydrophobic amino acids. The amino acids form circles due to the circular geometry of the c-ring. The first and the strongest circle is placed in the proximity of grooves between Leu 19 and Leu 15 residues, the second between Leu 15 and Ile 11 and the third between Ile 11 and Ala 7, respectively. In addition, there is electron density in the center of each circle (Fig. 1C). The distance between the circles of the densities corresponds to one turn of an α-helix or the distance between the strands of a β-sheet. However, the presence of a peptide inside the c-ring pore was not identified. Moreover, no part of either *a* or *b* subunit can fit the pore. Indeed, in accordance with recent cryo-EM structure^6^ both *a* and *b* subunits are located far from the interior of the c-ring to be able to deepen into it. The densities are not characteristic for DNA or RNA or for membrane lipids and therefore the electron densities can hardly be assigned to these molecules. These densities cannot arise from membrane lipid molecules that are often suggested to plug the c-ring pore (e.g.^29^). A single bond distance between C-C atoms of hydrocarbon chain is about 1.5 Å. However, the densities are placed at the distance of about 5.4 Å, which is approximately characteristic to that between the methyl groups of an isoprenoid chain molecule (~5 Å). An intriguing fact is that the densities have a universal character. Despite the fact that they were not recognized in previous studies, they are common for all the known high-resolution structures of mitochondrial, archaea, and bacterial c-rings (Table S2).

### Other unusual features of the internal pore of the c-ring

**Figure 2:**
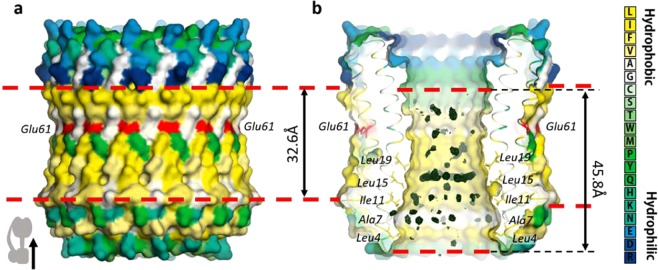
Van der Waals surface of the spinach chloroplast c_14_-ring with polar/apolar interfaces and additional electron densities. (**A**) The outer surface of the c-ring is shown. (**B**) Section clip of the c_14_-ring shows inner surface. Red dashed lines mark the putative positions of polar/apolar interfaces, which are different for inner and outer surfaces of the c-ring. The thickness of the apolar region is 45.8 Å and 32.6 Å for the inner and outer surfaces, respectively. Amino acids are colored according to their hydrophobicity and active centers Glu61 are colored red. The additional electron densities inside the c-ring are colored as deep green mesh, which shows the density map (Fo – Fc) at 3σ. Stroma of the chloroplast is on the top; thylakoid lumen is on the bottom of the picture.

First of all, the external and internal surfaces of the c-ring have a quasi-conical shape with wide parts at the membrane surface and narrow part in the center of the bilayer, close to Glu61 (Fig. 2). The inner diameter of stroma, central and lumen parts of the c-ring are 30 Å, 23 Å and 28 Å, respectively. It means that the lipids of lamellar bilayer, which prefer a cylindrical shape, can hardly fit to the shape of the pore. Second, a remarkable and previously not discussed feature is the unusually large hydrophobic thickness (45.8 Å) of the surface of the internal pore (according to PPM web server^30^). It is about 1.5 times larger than the hydrophobic thickness of the external part of the c-ring (32.6 Å) which is typical for integral membrane proteins and lipid bilayers. Third, the hydrophobic region of the pore is highly asymmetric relative to the center of the bilayer and Glu61 (Fig. 2). Fourth, it extends nearly to the surface of the stroma part of the c-ring and the size of a polar part is very small. Normally, the polar part of a membrane protein is no less than the thickness of polar part of its surrounding lipid bilayer (about 10 Å)^31^. It is the case of the external part of a hydrophobic/hydrophilic profile, but not of internal surface of the c-ring. All this add more reasons why usual membrane lipids can hardly fit the pore.

### Positive electron densities at the outside surface of the c-ring

**Figure 3:**
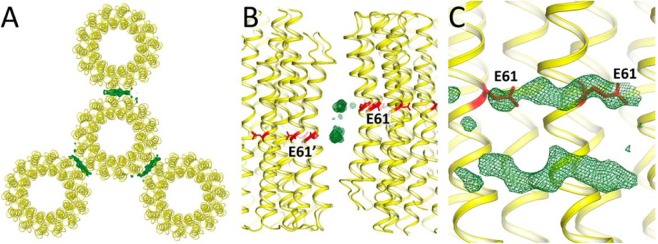
Crystallographic contacts between c_14_-rings in the crystal lattice. (**A**) View from the stroma of the chloroplast. C-rings from three neighbor asymmetric units are shown. Positive densities are present between each of two c-rings. (**B**) View between two c-rings in crystal. Active center (Glu 61) is colored in red. The additional density has two symmetric branches. Each branch contributes to its own C-ring, more likely to the one with the closest Glu 61. (**C**) View from outside on the c-ring. Two Glu 61 residues are close to the positive density. (Fo – Fc) difference electron density maps are shown at 3.4σ.

C subunits are well resolved in electron density maps and were easily assigned. However, there are additional and significant positive electron densities, which do not belong to the c-subunits. First, there is a short strip of narrow additional electron densities at the external surface of the rings covering Glu61, which is the key amino acid for generation of the c-ring rotation^6^, lying nearly parallel to the membrane plane (Fig. 3C). The densities are visible only in the places of the crystal contacts between adjacent c-rings oriented in opposite directions (Fig. 3A–C). The densities follow that part of an α-helix of the subunit a interacting with the c-ring in chloroplasts (Fig. S5)^6^. It is likely that each of two density strips is a part of one of the c-rings, more likely of the one with the closest Glu 61. We do not exclude that the densities are unfolded fragments of the α-helix 5 or 6 of a-subunit which in intact cF_1_F_O_ interact with the active site of the c-subunit^6^. We suggest that the densities may also correspond to another conformational state of the a-subunit corresponding to the fixed rotor/starter state. In other high-resolution c-ring structures there were no additional densities near their active centres. This might be connected with sample preparation or a crystallization method.

### Possible interpretation of unusual structural features of the c-ring

One of possible explanations is that characteristic electron densities may result from isoprenoid molecules covering internal surface of the c-ring pores. The isoprenoid quinones are natural components of these membranes known as electron and proton carriers to produce transmembrane proton gradients and existing in the same membranes as F_1_F_O_ ATP synthases. Therefore, *a priori* it is natural to consider them also as possible component of the interior of the c-ring. Thus, the structural data underline that the structure of the pore of the c-ring has unusual features pointing out on their possible functional significance.

**Figure 4:**
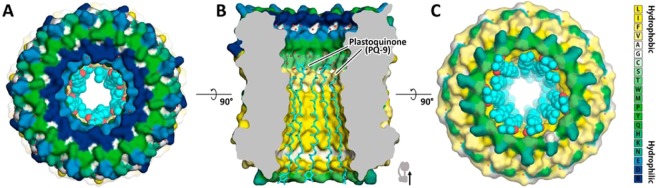
A schematic drawing showing the possible fit of inside of spinach c_14_-ring with plastoquinone (PQ-9) molecules. (**A**) View from stroma side. PQ-9 molecules are colored cyan and shown as spheres. (**B**) Side view of the c_14_-ring with hydrophobic area inside fitted with PQ-9 molecules. PQ-9 molecules are oriented in different directions and are shown with sticks. (**C**) View from lumen side. C-ring is shown in surface representation. The surface is colored according to the hydrophobicity of amino acids (see legend on the right).

Plastoquinone has a long hydrophobic isoprenoid chain, the number of isoprene groups n = 9 fits very well the large thickness of the hydrophobic interior of the c-ring (Fig. 4). Moreover, quinone isoprenoids such as plastoquinone, coenzyme Q (CoQ_10_. where **Q** refers to the quinone chemical group and **10** refers to the number of isoprenoid chemical units in its tail) and menaquinones are universal electron carriers in membrane respiratory and photosynthetic electron-transport chains of thylakoid, mitochondria and bacteria correspondingly. Thus, they coexist with ATP synthases in the same membranes.

This suggestion is also supported by the color of the crystals (Fig. S3) and the corresponding UV-Vis spectra showing characteristic for isoprenoids strong absorption of light in the range between 400 and 500 nm (Fig. S6A). In the work^32^ similar crystals of the c-ring of spinach ATP synthase were obtained. They also had a strong yellow color as our crystals. The authors^32^ performed spectroscopic and HPLC analyses of the dissolved crystals and concluded that carotenoids and chlorophyll molecules bounded to the c-ring could explain the color and fit the HPLC results of the dissolved crystals. Moreover, considering that the protein preparation was done under harsh detergent and temperature conditions (sodium lauroyl sarcosinate 1% w/v and 65 °C) they supposed that these molecules “may be located inside the c-ring”^32^.

The spectra in Fig. S6A show that the peak of absorption at 335 nm from our c-ring samples and dissolved crystals of the c-ring from^32^ cannot be explained by beta-carotene or chlorophyll *a* molecule, but, for example, neutral plastosemiquinone-9^33^ or its derivatives might be one of the candidates. The qualitative reaction with sodium borohydride (NaBH_4_) for reducing ketones and aldehydes was done with the c_14_-ring samples dissolved in EtOH and showed different spectra for the samples with and without NaBH_4_ (Fig. S6B). Differential UV-Vis spectrum of the c_14_-ring samples dissolved in EtOH without (oxidized form) and with (reduced form) NaBH_4_ has a similar peak at 260 nm as a trimethyl benzoquinone (TMBQ), that is a polar moiety of plastoquinone-9 (Fig. S6D–F).

**Figure 5:**
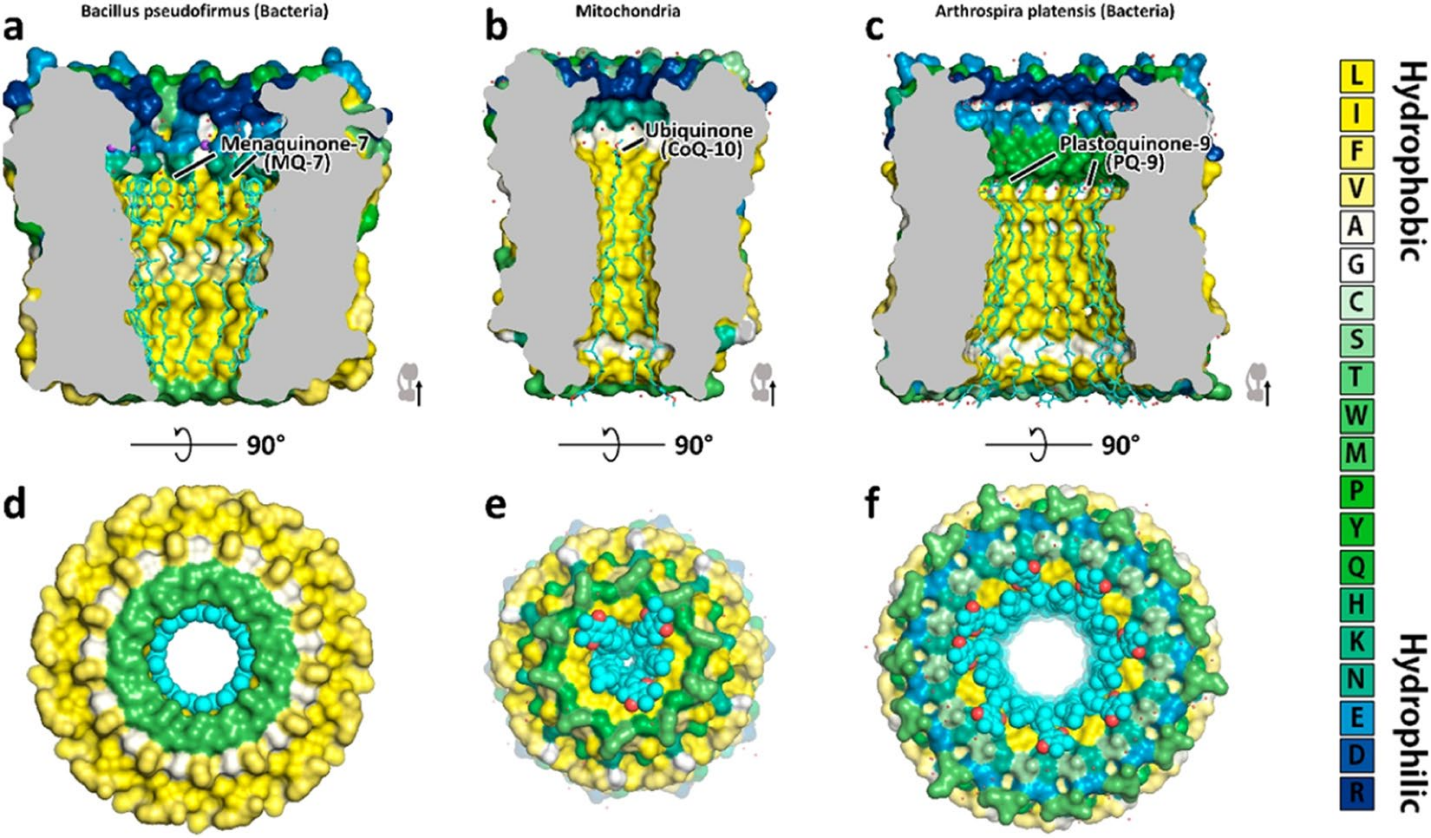
Suggested schematic drawing showing the possible fit of inner part of c-rings from different organisms with corresponding quinone molecules inside. (**A**,**D**) – c-ring from *Bacillus Pseudofirmus* with menaquinone-7 (MQ-7) inside. (**B**,**E**) – c-ring from yeast mitochondria with ubiquinone-10 (UQ-10). (**C**,**F**) **–** c-ring from *Arthrospira platensis* with plastoquinone-9 (PQ-9). MQ-7, UQ-10 and PQ-9 are electron carriers in *Bacillus Pseudofirmus*, yeast and plants, respectively. The molecules are oriented in different directions and shown in sticks. C-rings are shown in surface representation. The surface is colored according to the hydrophobicity of amino acids (see legend on the right).

Interesting is that the thickness of the hydrophobic part of internal c-ring surface in thylakoid, mitochondrial and bacterial ATP synthases (Figs. S7–S9) is correlated quite well to the lengths of hydrocarbon chains of isoprenoid quinones (plastoquinones, coenzyme Q and menaquinones) found in the corresponding membranes comprising photosynthetic and respiratory electron transport chains. Interestingly, isoprenoid quinones into the structures fit the interior of different c-rings (Figs. 4 and 5).

## Discussion

First of all, the c-ring structure itself is important for two reasons. This is the first and the only high-resolution structure of c-rings from plants and this was the missing part in the picture of the high-resolution structures that were obtained from mitochondria and bacteria. The high-resolution structures of mitochondria and bacterial c-rings are already known. The fact that chloroplast c-rings are similar to that from mitochondria and bacteria is important. The second reason is that the structure of the c-ring revealed molecular mechanisms of intersubunit contacts which determine the size of the c-ring (Figs. S13–15). Although this result was discussed previously in literature^1,2,28^ and the fact that the c-ring from spinach chloroplasts has 14 subunits was also known^22^, in this work due to high-resolution crystallographic data we showed in details the network of hydrogen bonds, compared the intersubunit contacts of c-rings from plants, mitochondria and bacteria, and showed molecular mechanisms of how determination of c-ring stoichiometries occurs.

We described a striking feature of the structure of the c-ring ATP synthase from chloroplasts – the presence in the hydrophobic part of the internal pore of the c-ring of circular-like electron densities placed at the distance of about 5.4 Å from each other along the central axis and parallel to the membrane plane. As we stressed, the present observation becomes even more important due to the fact (as it appears) that it is a universal feature of all known high-resolution structures of other F_1_F_O_ synthase c-rings.

We present the evidences that the densities might correspond to isoprenoid quinones. It is consistent with about 1.5 times larger hydrophobic thickness of hydrophobic surface of the inside of the c-ring than that of a lipid bilayer and a membrane protein. UV-Vis spectra (Fig. S6A) comprise isoprenoids presumably inside the c-ring and differential UV-Vis spectroscopy shows quinones presumably with trimethyl benzoquinone (TMBQ) (PQ-9 polar moiety) in the c-ring samples dissolved in EtOH (Fig. S6D–F). Although the noise is quite high in the differential spectrum of the c-ring samples (Fig. S6D) (about 0.01 a.u. of absorption) the peak at 260 nm can be clearly seen and the whole curve is close to the TMBQ differential spectrum. The high noise might be due to the low absolute concentration of the compound reacting with NaBH_4_ because of the high dilution ratio of the c-ring samples in EtOH (~1:30). However, that was done to eliminate changes in the solvent (EtOH) caused by the buffer in which the c-ring protein is and for proper control experiments.

The schematic drawings showing possible fit of different isoprenoid molecules (Figs. 4 and 5) into c-rings from different isoprenoids also point towards the hypothesis. Finally, the universality of the additional densities inside different c-rings (Figs. S7–S9) suggest that the molecule inside the c-ring should have universality among different species, long hydrophobic tail, contain isoprenoids and be wide-spread among different species. Thus, taking into account the harsh conditions of preparation (+65 °C, NLS 1%) of the c-ring for the UV-Vis studies we conclude that the isoprenoids, in particular, plastoquinone or its derivatives might be one of the candidates. Moreover, since we do not see any fragmented electron densities of an isoprenoid molecule at the external surface of the c-ring we suggest that isoprenoids might be inside of the c-ring.

To our best knowledge this hypothesis since 2008^32^ was not furthermore discussed and developed. There was no proof that carotenoids are inside of the c-ring pore. Indeed, the detergent treatment of membrane proteins does not remove (at least completely) native lipids surrounding the protein surface. An example of this is a squalene molecule at the hydrophobic surface of bacteriorhodopsin evidenced with high resolution crystallographic structures obtained with the crystals grown from the protein solubilized in a quite harsh detergent beta-octyl-glucoside^34^.

Our crystallographic data do not provide an evidence of the presence of isoprenoid molecules at the surface of the c-ring, in opposite, as we have shown, the characteristic for isoprenoid molecules electron densities are present inside the c-ring. Taking into account that usually only tightly bound amphiphilic molecules remain bound to a membrane protein and are simultaneously resolved on electron density maps we conclude that the molecules responsible for the color of the crystal are really placed inside the c-ring.

The color of the crystals is determined by absorption of light in the range of 400–500 nm. It is a characteristic of carotenoids, however, contribution to the absorption range is also characteristic of some other isoprenoids. Unfortunately, the analysis of the spectra is even more complicated due to the possible presence of chlorophyll molecules in the examined samples.

Thus, despite the fact that electron densities inside the c-ring, spectroscopic and HPLC data point toward the presence of an isoprenoid molecule inside the c-ring, all this itself is not sufficient to identify the exact type of the molecule.

In general, identification of lipid molecules on electron density maps of membrane proteins is a challenge. In the most cases the densities related to the lipids are weaker, fragmented and usually polar heads are not resolved at all^35^. However, in the present case there are hints, which make the identification more reliable. The unusually large thickness of the hydrophobic surface of the internal part of the c-ring means that the lipids should have unusually long hydrophobic chains. Carotenoids first emerged in archaebacteria as lipids reinforcing cell membranes. To serve this function their linear chain is comprising usually 9 to 11 conjugated C = C bonds with the length corresponding the thickness of the hydrophobic part of lipid membranes (30–35 Å). Thus, they alone cannot explain the hydrophobic thickness of the pore of the c-ring. Moreover, carotenoids are not found in mitochondria and therefore they can hardly explain similar electron densities in yeast mitochondria ATP-synthases c-rings (Fig. S6). In opposite, other polyisoprenes may fit the requirements. The best known of such polyisoprenes are ubiquinone in the mitochondrion and in prokaryotes^36^, and plastoquinone (PQ) and similar isoprenes are associated with the chloroplast and prokaryote photosynthetic membranes^37^.

We note that Meier *et al*. suggested that in their experiments^29^ phospholipids are incorporated into the central hole from one side of the cylinder during the reconstitution procedure, as the detergent-purified c-ring is completely devoid of phospholipids. The authors recognize that “the association with phospholipids during the reconstitution procedure merely indicates a strong affinity of the C_11_ cylinder for hydrophobic molecules to its central cavity” Thus, Meier *et al*. support the idea of high affinity of c-ring for hydrophobic molecules to its central pore. Isoprenoids are specific, but also very hydrophobic lipids. Therefore, we should also note that a mixture of different lipids, for instance isoprenoids and phospholipids, may fill better the central pore of the c-ring. In addition, we have indirect evidence on UV-Vis spectra (Fig. S6) that there might be a mixture of different lipids and isoprenoids due to absorption peaks corresponding to beta-carotene and chlorophyll *a* molecules together with absorption peak at 335 nm, that does not correspond to beta-carotene or chlorophyll *a*, however might correspond to plastosemiquinone-9 or its derivatives^33^.

It is noted that additional electron densities in the internal part of the c-rings are not equally strong in all other known high-resolution structures of c-rings. However, there is a strong correlation between the level of additional electron densities inside the c-rings and their purification and crystallization conditions (Table S3). The structures obtained from intact ATP synthases, without using harsh conditions (NLS or/and high temperature) demonstrate pronounced additional characteristic electron densities in the inner pore (4F4S and 5BPS, Fig. S7). The c-rings for which harsh solubilization and purification conditions were used demonstrate less pronounced additional electron densities (4CBK and 2 × 2 V, Fig. S8). The only exception is the structure 2XQU, where well-ordered circles of additional electron densities take place, though harsh conditions were used to purify the c-ring (Fig. S9B).

A unique arrangement of electronic levels due to the polyene chain structure makes isoprenoids also efficient protectors of the cell against reactive oxygen species (ROS)^38^. The importance of quinone isoprenoids is not limited by the electron carrier functions, for instance, coenzyme Q is an essential component of the respiratory chain, a cofactor of pyrimidine biosynthesis, a proton permeability barrier^39,40^ and acts as an antioxidant in mitochondrial membranes. More recently CoQ has been identified as a modulator of apoptosis, inflammation and gene expression^38^.

Thus, we speculate that isoprenoid quinones, which present in membranes with F_O_F_1_ ATP synthases may also present inside the c-rings. We suggest that they may play an important role as scavengers of ROS protecting not only external but also internal part of the c-rings, in particular against ROS. This is supported also by the fact that, in opposite to carotenoids, in vertebrates these molecules damaged by ROS are synthesized *de novo*^41^. In addition, by the studies of BCDO2-deficient mice and human cell cultures it indicates that carotenoids can impair respiration and induce oxidative stress. Mammalian cells thus express a mitochondrial carotenoid-oxygenase that degrades carotenoids to protect these vital organelles^42^.

We hypothesize that isoprenoid quinones, such as plastoquinone and coenzyme Q, may be universal cofactors of proton ATP synthases and their abnormalities may lead to cell and tissue dysfunctions not only through disturbance of the electron transport bioenergetics chain but also directly through dysregulation of ATP synthases. Although this hypothesis seems plausible, we recognize that additional experiments are necessary to verify it and other possible explanations should be considered. However, precise identification of what molecule corresponds to the electron densities inside the c-ring is possible only by high-resolution crystallography. It is a well-known problem in case of lipids bound to a membrane protein. Nevertheless, what is indeed clear is that inner part of c-rings of different F_1_F_O_ ATP-synthases have universal unusual features, which may have a great functional importance, and isoprenoid quinones correspond well to obtained experimental data and their universality among different organisms.

It is not excluded that not only isoprenoid molecules could be placed inside c-ring but also in addition there might be some of F_O_ subunits of ATP synthase partially entering the inner pore of the c-ring. Indeed, works^8,10,43^ show that in case of mitochondrial ATP synthases C terminus of F_O_ subunit *e* might be extended to the center of the c-ring. Moreover, in c-rings of V-type ATP synthases^44,45^ the densities inside were assigned to two alpha helices. The work^44^ also shows that one of the alpha helices placed in the center of the pore belongs to V_O_ part of the V-type ATP synthase. We speculate that the presence of an anchor in the center of the c-ring may have functional importance. Namely, one cannot exclude that this internal anchor is connected with a subunit of the stator F_O_ part of ATP synthase. This would additionally stabilize precise positioning of the rotor to the stator in the region of proton transfer pathway (active site of the c-ring), (Fig. S10), similarly as in electrical motors, where the stator and rotor precise positioning is absolutely necessary. Reviews^1,2^ mention surprisingly small contact between *a* subunit and c-ring and it can be not sufficient to stabilize positioning of Glu61 active site relative to *a* subunit. Thus, we hypothesize that isoprenoid layer covering inner surface of the c-ring (Fig. 5) may also reduce frictions between the anchor of the stator and the surrounding upon rotation of the c-ring (Fig. S10).

Due to the variations of the number of *c* subunits in the c-ring (8 to 17), the diameters of c-rings vary significantly (Table S4), which leads to the noticeable difference in the angle between interacting c-subunits. Consequently, the distances between outer helices of the c-ring vary also significantly, especially in case of their edges (Fig. S14). Thus, it is essential that in smaller c-rings (c_9_, c_10_) interactions of the outer helices are weaker than in case of large c-rings (c_14_, c_15_), while interactions between inner helices is expected to be tighter in case of smaller c-rings. This explains the absence of any conserved hydrogen bonding between the outer helices of neighbor subunits of different c-rings, except the active center region. The glutamate sidechain of the active center is a part of the oligomerization interface in all bacterial, mitochondrial and chloroplast ATP synthases. However, in large c-rings glutamate side chain is bonded directly to the backbone of the neighbor subunit (Fig. S15A,B), while in the small c-rings this connection is mediated by water molecules (Fig. S15C,D). This results in the increase of the distance between outer helices in case of c_9_ and c_10_ rings. For the inner helices there is the opposite situation. Like in case of chloroplast c-ring, dense hydrogen bonding between inner helices occurs close to F_1_ side of the c-ring in the hydrophilic region due to the presence of polar amino acids, comprising this part of the c subunit. This bonding is usually mediated by several water molecules (Fig. S15). Another common feature is the presence of additional stabilizing hydrogen bonds at the level of the active center in the inner side of the c-rings. But in case of smaller c-rings the connection is tighter, because the water molecule, which mediates the hydrogen bonding in this region, interacts directly with the oxygens and nitrogens of the main chains of neighbor subunits. This leads to the closer positioning of inner helices in case of smaller c-rings. The motifs described in^28^ are important for c-rings stoichiometry and we found experimentally how these interactions between c-subunits determine the c-ring size (e.g. the number of c-subunits in the c-ring).

## Materials and Methods

### Purification of intact and active CF_1_F_O_

Chloroplast ATP synthase was isolated from fresh Spinach leaves (*Spinacia oleracea L*.) according to^20,21^. Chloroplast membranes were solubilized with sodium cholate (23 mM) and octyl-β-D-glucopyranoside (55 mM). The ATP-synthase was partially separated from lipid and contaminating proteins by fractionated ammonium sulfate precipitation and rate zonal centrifugation. The medium contained azolectin, 1 mg/mL, and n-dodecyl-β-D-maltoside (DDM), 8 mM (step gradient containing 15%, 22%, 29%, 36%, 43%, 50% sucrose). After centrifugation at 134 000 g for 14 h (Beckmann VTI 50 rotor) CF_O_F_1_ was found in the 36% and 43% sucrose fraction. Subsequently, the enzyme was desalted by passage through a HiTrap Desalting Column and further purified by dye-ligand chromatography employing reactive red 120^46^.

### Electrophoresis

Blue Native PAGE was performed using the Hoefer Mighty Small II SE 250 system (small gel: 10 × 8 × 0.15 cm^3^) as described in^20,46,47^. The stacking gel had an acrylamide concentration of 3% and the separating gel acrylamide gradients from 3.5% to 16%. Thirty micrograms of solubilized purified cF_1_F_O_-ATP synthase in DDM was loaded per lane. A high molecular weight native marker (GE Healthcare) served as mass standard. After gel run the BN-Gel was stained additionally with Coomassie R-250.

SDS-PAGE was performed according to^48^, with a stacking gel of 3% and separating gel of 14%. In order to maintain integrity of the subunit *c* oligomer, protein sample were incubated at room temperature in SDS loading buffer for 10 min. After electrophoresis the gel was stained by Coomassie (Fig. S2A,B).

### Quantification of ATP generation energized by electrochemical gradient

Reconstitution of purified ATP-synthase into liposomes (phosphatidyl choline/phosphatidic acid, 9:1, w/w) was performed according to^49^ with slight modifications. The mixture of solubilized cF_1_F_O_, lipids and detergents (sodium cholate, sodium deoxycholate, 800 µL) was dialyzed against 800 mL of reconstitution buffer containing 10 mM Tricin, 0.2 mM EDTA, 10 mM MgCl_2_, pH 8.0, 0.25 mM DTT for at least 7 hours at 4 °C. Inhibition of cF_1_F_O_ by dicyclohexylcarbodiimid (DCCD, 50 µM, added before reconstitution into liposomes and incubated for 30 min at room temperature) served as a control.

The activity of ATP-synthesis was determined as previously described^50,51^. 240 µL buffer L2 (200 mM Tricin, 5 mM sodium dihydrogen phosphate, 120 mM KCl, 2.5 mM MgCl_2_, 0.2 mM ADP) was added into a clinicon-cuvette and mixed with 12.5 µL Luciferin-Luciferase-reagent. The cuvette was placed into a Luminometer (1250, BioOrbit) and the baseline was recorded. 43 µL of proteoliposomes were equilibrated with 217 µL buffer L1 (20 mM sodium succinate, 5 mM sodium dihydrogen phosphate, 2.5 mM MgCl_2_, 0.6 mM KCl, 1 µM Valinomycin, pH 4.7). After 100 seconds the mixture was injected via a cannula into the cuvette containing L2. ATP-synthesis driven by the established transmembrane electrochemical gradient (ΔpH and ΔK^+^) was monitored as luminescence applying the Luciferin-Luciferase-Assay. To determine the activity, the initial slope of the measured signal was assessed. The luminescence signal was calibrated by addition of 20 µL 10 µM ATP. For further information please refer to Fig. 1C in^13^.

### Protein crystallization

The crystals were grown using the *in meso* approach^52,53^, similarly to our previous works^24,54^. The solubilized protein in the crystallization buffer was added to the monooleoyl-formed lipidic phase (Nu-Chek Prep, USA). The best crystals were using protein concentration of 27.4 mg/mL and the 0.1 M MES pH 5.8, 1.4 M ammonium sulfate precipitate solution from the Qiagen Cubic Phase I screening kit (Qiagen, Germany). Crystallization trials were set up using the NT8 robotic system (Formulatrix, USA). The crystals were grown at 22 °C. Regular shaped cubic crystals reaching 20 µm size appeared in approximately 2 months and had a strong yellow color (Fig. S3).

### Acquisition and treatment of diffraction data

X-ray diffraction data were collected at the beamline ID23-1 of the ESRF, using a PILATUS 6 M detector. Diffraction patterns were integrated using XDS^55^. The space group was determined to be I121. The reflexes’ intensities were scaled and merged using the AIMLESS software from the CCP4 program suite^56^. The data statistics is presented in Table S1.

### Structure determination and refinement

Initial phases were successfully obtained in the I121 space group by a molecular replacement (MR) method using MOLREP^57^ with the model 3V3C^12^. The initial MR model was then iteratively refined using REFMAC5^58^ and Coot^59^.

### UV-Vis differential spectroscopy

UV-Vis spectroscopy studies were done using the spectrophotometer Shimadzu UV-2450. The c_14_-ring samples were dissolved in EtOH for analysis of their pigments compound. The qualitative reaction with sodium borohydride (NaBH_4_) was done. The reaction reduces ketones and aldehydes to alcohols and changes their absorption spectra. In case of quinones they can be reduced to quinols (with two OH groups instead of oxygens) or semiquinones (one OH group and one oxygen). We measured the c_14_-ring samples dissolved in EtOH (oxidized form) in ratio ~1:30 (v/v), then added several grains of NaBH_4_ on the tip of a spatula (reduced form) and incubated 2 min. The measurements range was from 230 to 750 nm. Two spectra were subtracted (ox - red) (Fig. S6B,D) and compared with a «UV-Vis» structural analogue of plastoquinones trimethyl benzoquinone (TMBQ) that is a polar moiety of plastoquinone-9. TMBQ dissolved in EtOH was measured with and without NaBH_4_ and its UV-Vis differential spectrum was compared with the c_14_-ring samples (Fig. S6D). EtOH with and without NaBH_4_ was measured as a control experiment and showed a negligible difference between two spectra (Fig. S1C).

## Supplementary information

Unusual_features_of_the_c-ring_of_F1FO_ATP_synthases_SI

## Data Availability

All data is available in the main text or the supplementary materials.
