# Supplementary material for: Unusual features of the c-ring of F1FO ATP synthases
Source: Sci Rep. 2019 Dec 6;9:18547. doi: 10.1038/s41598-019-55092-z (PMC6897951; doi:10.1038/s41598-019-55092-z)
Supplement: Supplementary file 1 — Unusual_features_of_the_c-ring_of_F1FO_ATP_synthases_SI [file 41598_2019_55092_MOESM1_ESM.docx]

Unusual features of the c-ring of F_1_F_O_ ATP synthases

A.V. Vlasov^†1,4^, K.V. Kovalev^†1,2,3,4^, S.-H. Marx^†5^, E. Round^†2^, I. Gushchin^1,3^, V. Polovinkin^2^, N.M. Tsoy^1˟,6^, I.S. Okhrimenko^1^,V.I. Borshchevskiy^1^, G. Büldt^1^, Yu.L. Ryzhykau^1^, A.V. Rogachev^1,7^, V.V.Chupin^1^, A.I. Kuklin^1,7^, N.A. Dencher^1,5^ and V.I. Gordeliy^2,3,1^*

^1^Research Center for Molecular Mechanisms of Aging and Age-Related Diseases, Moscow Institute of Physics and Technology, Dolgoprudny, Russia.

^2^Institut de Biologie Structurale Jean-Pierre Ebel, Université Grenoble Alpes–Commissariat à l’Energie Atomique et aux Energies Alternatives–CNRS, Grenoble, France.

^3^Institute of Complex Systems (ICS), ICS-6: Structural Biochemistry, Research Centre Jülich, Jülich, Germany.

^4^Institute of Crystallography, RWTH Aachen University, Aachen, Germany.

^5^Physikalische Biochemie, Fachbereich Chemie, Technische Universität Darmstadt, Alarich-Weiss-Straße 4, D-64287 Darmstadt, Germany.

^6^Department of Biochemistry, University of Zurich, Zurich, Switzerland.

^7^Joint Institute for Nuclear Research, Dubna, Russia.

*Correspondence to: valentin.gordeliy@ibs.fr

^†^equal contributions

^˟^affiliation during working on this manuscript

Supplementary Materials

**The structure of the active center (surrounding of Glu61)**

The conformation of the active center corresponds to “closed” (protonated) form which is also the case of almost all structures of *c* rings published^14–16,18,19^. The difference between the “closed” and “opened” (protonated and deprotonated) forms is in conformation of the active center, specifically in the conformation of glutamate amino acid, which can rotate through an angle of 95° around C – C bond. In literature, there is only one example of a high-resolution structure of the c_10_-ring in the opened state (3U2F)^17^ which relates to the c-ring from *Saccharomyces cerevisiae*. In Fig. S11A, B we show an alignment of our and 3U2F structures of the active centers. The only difference is the orientation of Glu amino acid (Fig. S11B). Comparison of active centers of our and other high-resolution structures of c-rings is shown in Fig. S12. In chloroplast c_14_-ring Glu61 side chain is well-stabilized by hydrogen bonding with Gln28, Tyr66’ side chain and Phe59’ oxygen. Very similar conformation was observed in case of bacterial *c_15_*-ring from *Arthrospira platensis* (PDB: 2XQU). However, in most cases glutamate side chain of the active center is stabilized weaker with hydrogen bonding with neighbor c-subunit backbone via water molecule (Wat201 in c_10_-ring of yeast mitochondria, PDB: 5BPS; and Wat2006 in c_13_-ring of bacteria *Bacillus pseudofirmus*, PDB: 2X2V, 4CBK).

**Table S1.** Data collection and refinement statistics of spinach chloroplast ATP-synthase *c*_14_ ring.

| **Data collection** |  |
| --- | --- |
| Space group | I 1 2 1 |
| ***Cell dimensions*** |  |
| *a*, *b*, *c* (Å) | 93.14, 96.34, 158.68 |
| *α, β, γ* (°) | 90, 106.72, 90 |
| Wavelength (Å) | 0.979 |
| Resolution (Å) | 48.17-2.30 (2.36-2.30) |
| *R*_merge_ (%) | 19.2 (116.9) |
| *R*_pim_ (%) | 15.7 (92.6) |
| *I*/*σI* | 4.9 (1.2) |
| *CC1/2 (%)* | 99.3 (42.1) |
| Multiplicity | 3.7 (3.5) |
| Completeness (%) | 96.7 (75.6) |
| Unique reflections | 57,691 (3484) |
| **Refinement** |  |
| Resolution (Å) | 20-2.30 |
| No. reflections | 54,827 |
| *R*_work_/ *R*_free_ (%) | 21.0/24.5 |
| ***No. atoms*** |  |
| Protein | 7935 |
| Water | 15 |
| Lipid fragments | 132 |
| ***B-factors (Å^2^)*** |  |
| Protein | 30 |
| Water | 34 |
| Lipid fragments | 48 |
| ***R.m.s deviations*** |  |
| Protein bond lengths (Å) | 0.0056 |
| Protein bond angles (°) | 0.8712 |
| ***Ramachandran analysis*** |  |
| Favored (%) | 97.5 |
| Outliers (%) | 0 |


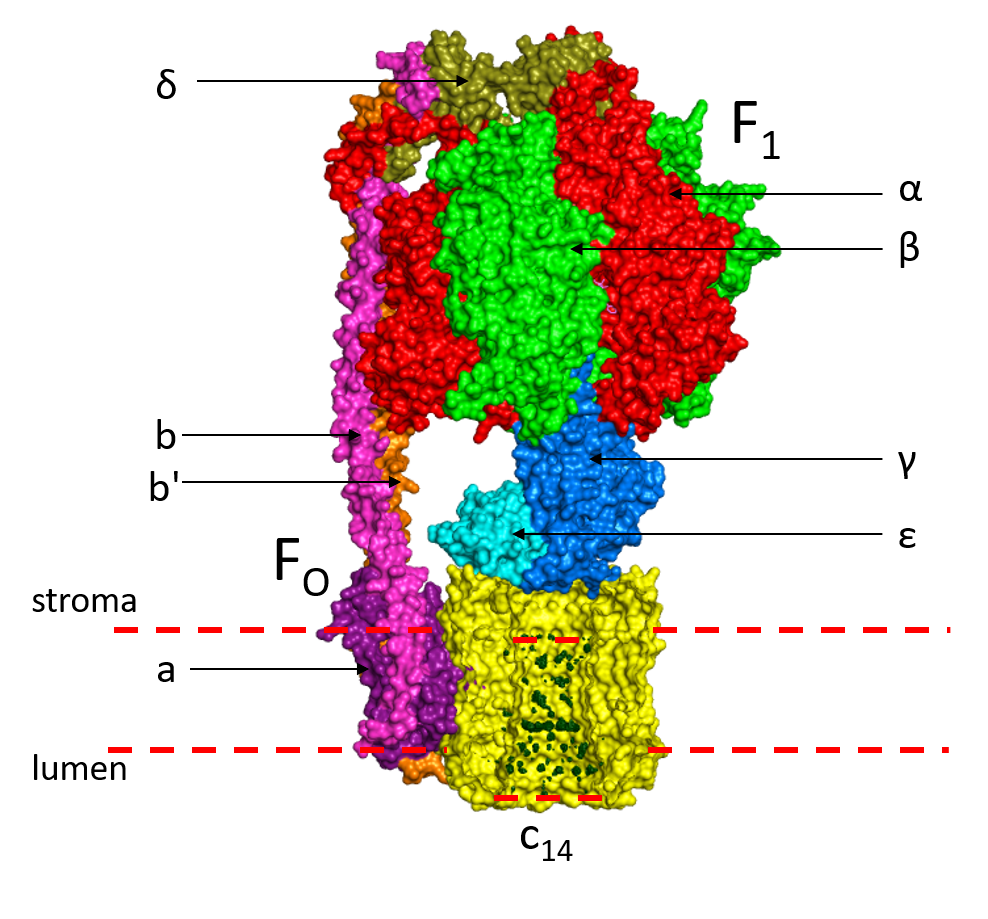


**Fig. S1.** The mosaic model of spinach chloroplast H^+^ cF_O_F_1_ ATP synthase. The model was built using F_O_F_1_ part from 6FKF^6^ and our structure of the c_14_-ring.

**A B**


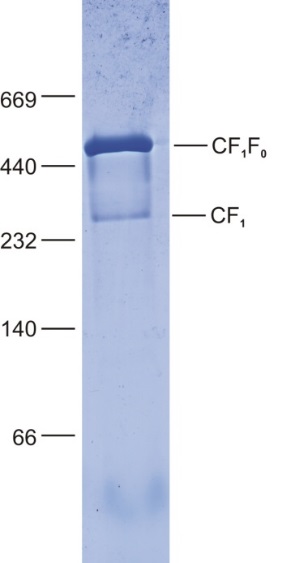

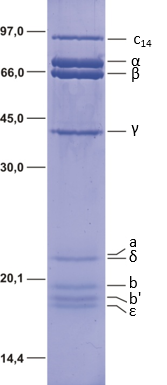


**Fig. S2.** BN gel Coomassie R-250 stained of 30 μg CF_1_F_O_-ATP synthase isolated from spinach chloroplasts; **(A)** high yield of intact enzyme, only a small amount of CF_1_. **(B)** SDS gel of the purified CF_1_F_O_-ATP synthase from spinach chloroplasts. The gel reveals the F_1_ subunits α_3_β_3_γδε and the F_O_ subunits *a*, *b*, *b’* and *c*. Subunit *c* migrates as a 14-protomer entity. Subunit *a* has a faint lane that might be due to the composition of amino acids of fully membrane subunit.


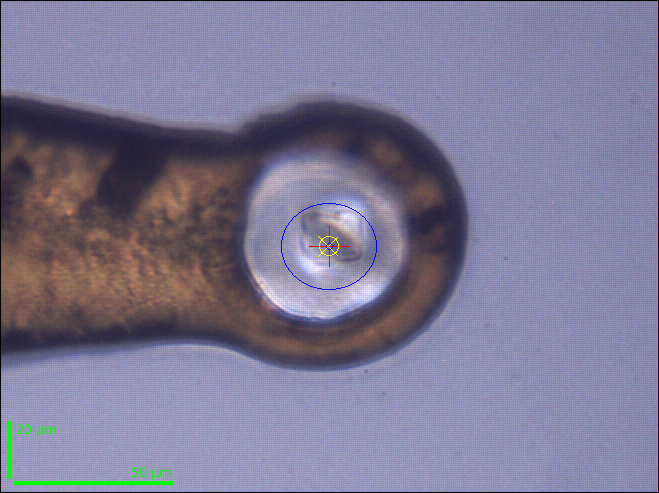


**Fig. S3.** Photo of the spinach c-ring crystal. The photograph of the c-ring crystal that was used for data collection. The crystals have a strong yellow color.


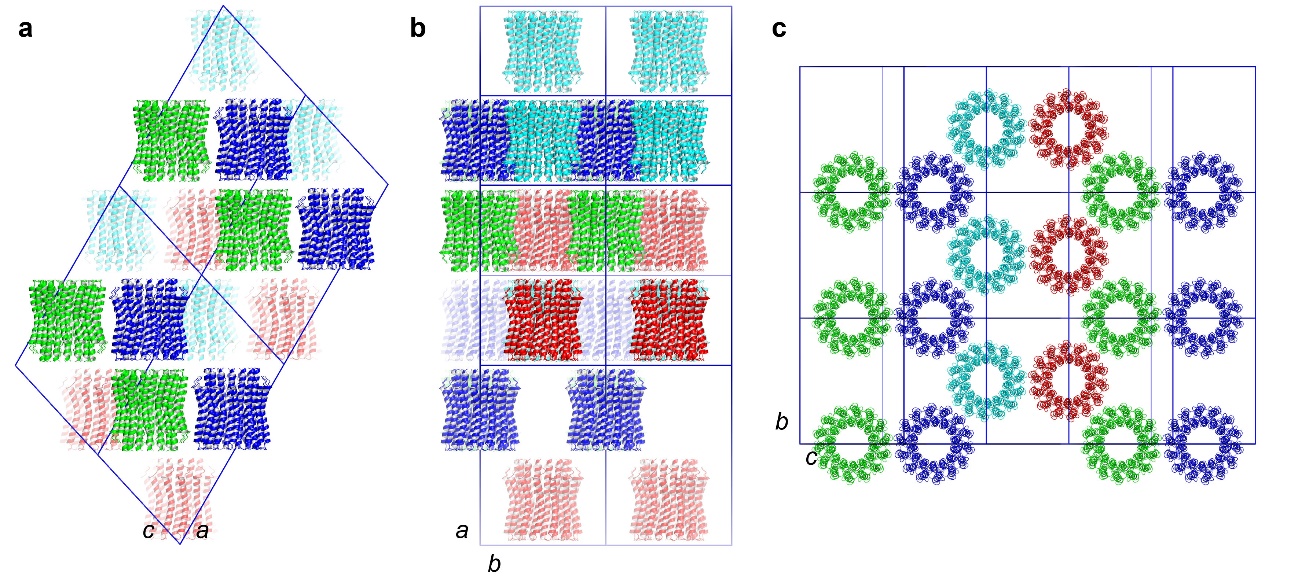


**Fig. S4.** Crystallization packing of the c_14_-ring from spinach chloroplast and its crystallographic contacts. The symmetry is I121 space group with the angles alpha = 90.0, beta = 106.7, gamma = 90.0 and projections on (**A),** a/c **(B),** a/b **(C),** b/c plane.


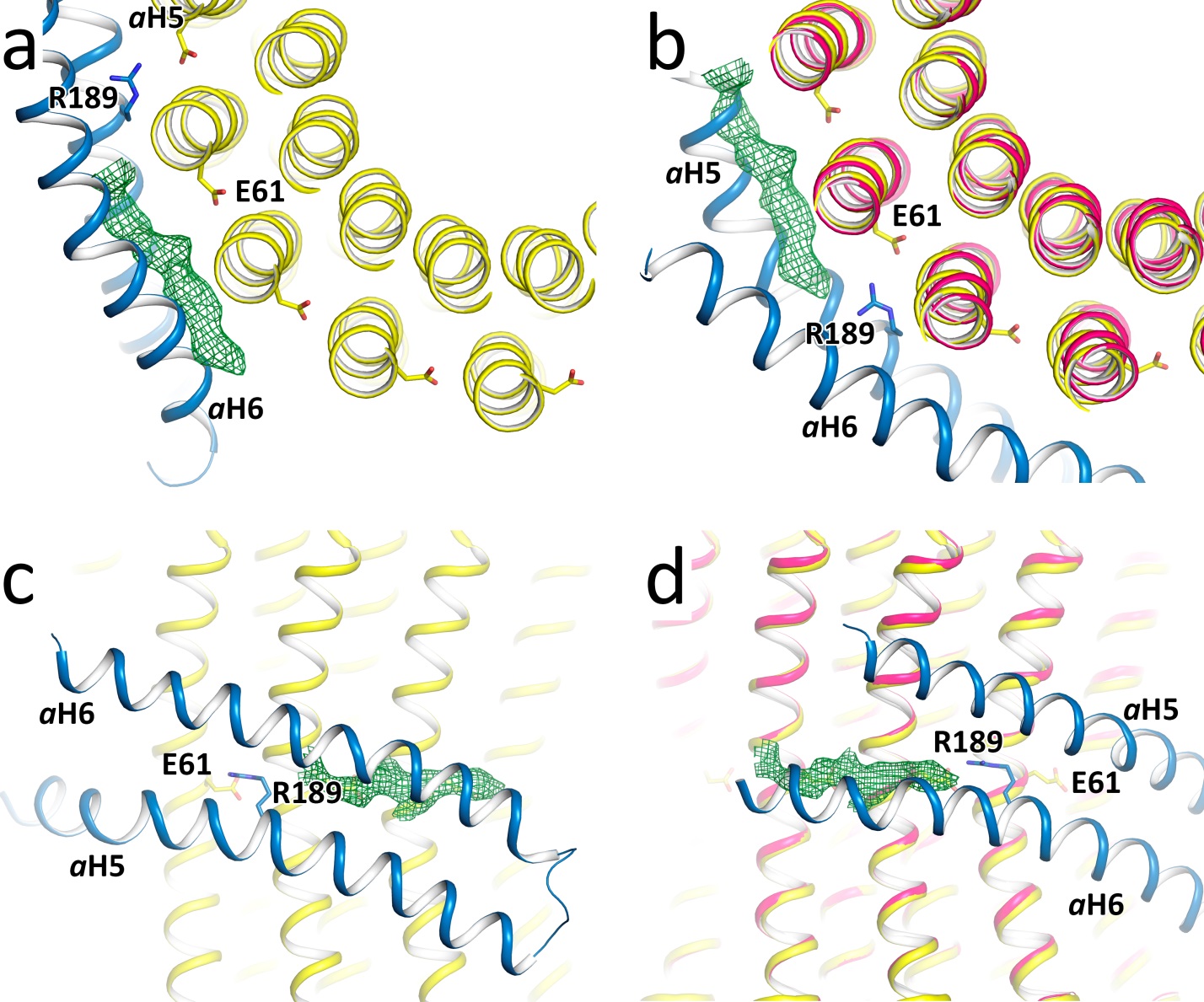


| **Fig. S5.** Positive difference electron densities outside of the c-ring and possible fit with *a* subunit from 6FKF model of chloroplast ATP synthase (helices 5 and 6 of a subunit are shown in blue). **(A), (C)**. Fit with the helix 6. **(B), (D)**. Fit with the helix 5. Fo-Fc difference electron density maps are colored green and are contoured at the level of 3.0σ. 6FKF model was aligned to chloroplast C ring model from this work only by C ring part. |
| --- |

**
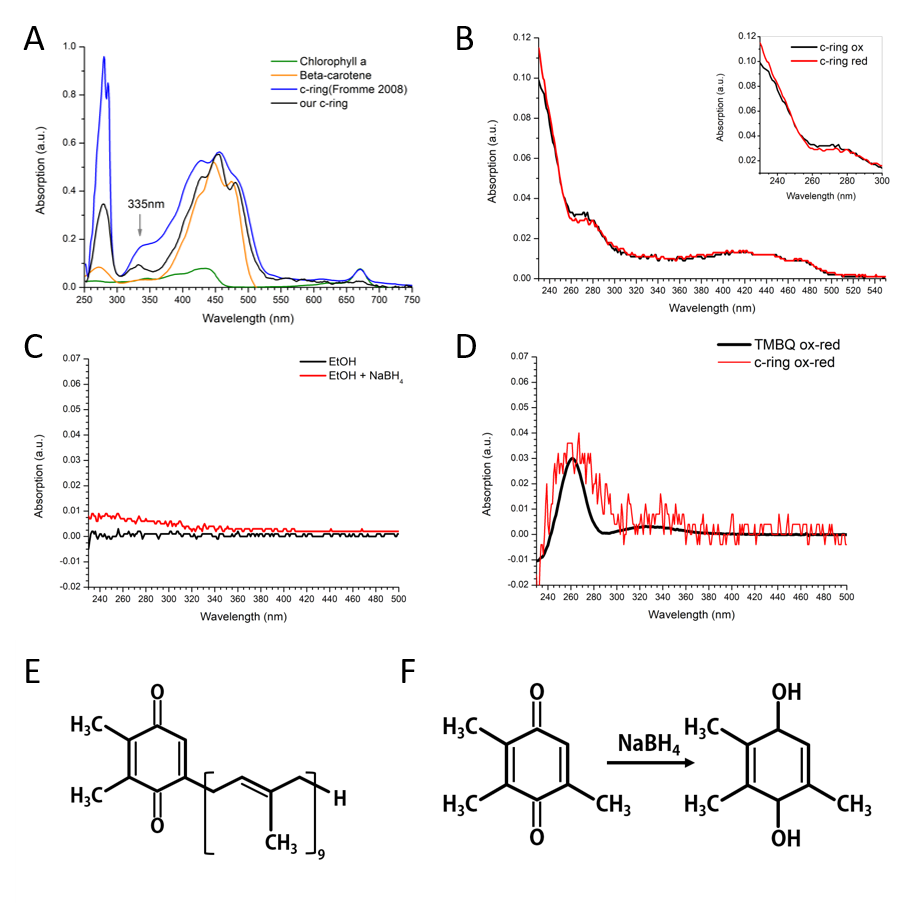
**

**Fig. S6.** UV-Vis spectral analysis of c-ring samples. **(A)** Comparison of spinach chloroplast c-rings spectra. UV-Vis spectra of our c_14_-ring (black line) and dissolved crystals of spinach chloroplast c_14_-ring^31^ (blue line). For comparison the spectra of beta-carotene and chlorophyll *a* are shown (orange and green line, respectively). **(B)** UV-Vis spectra of the c_14_-ring dissolved in EtOH (oxidized form) and with addition of sodium borohydride (NaBH_4_) (reduced form) (black and red line, respectively). **(C)** Control experiment with EtOH and with addition of NaBH_4_ (black and red line, respectively). **(D)** UV-Vis differential spectra (oxidized minus reduced form) (ox-red) of c_14_-ring dissolved in EtOH and trimethyl benzoquinone (TMBQ) (red and black line, respectively). **(E)** plastoquinone-9 (PQ-9) and **(F)** reducing reaction of TMBQ (PQ-9 polar moiety) by NaBH_4_.

**
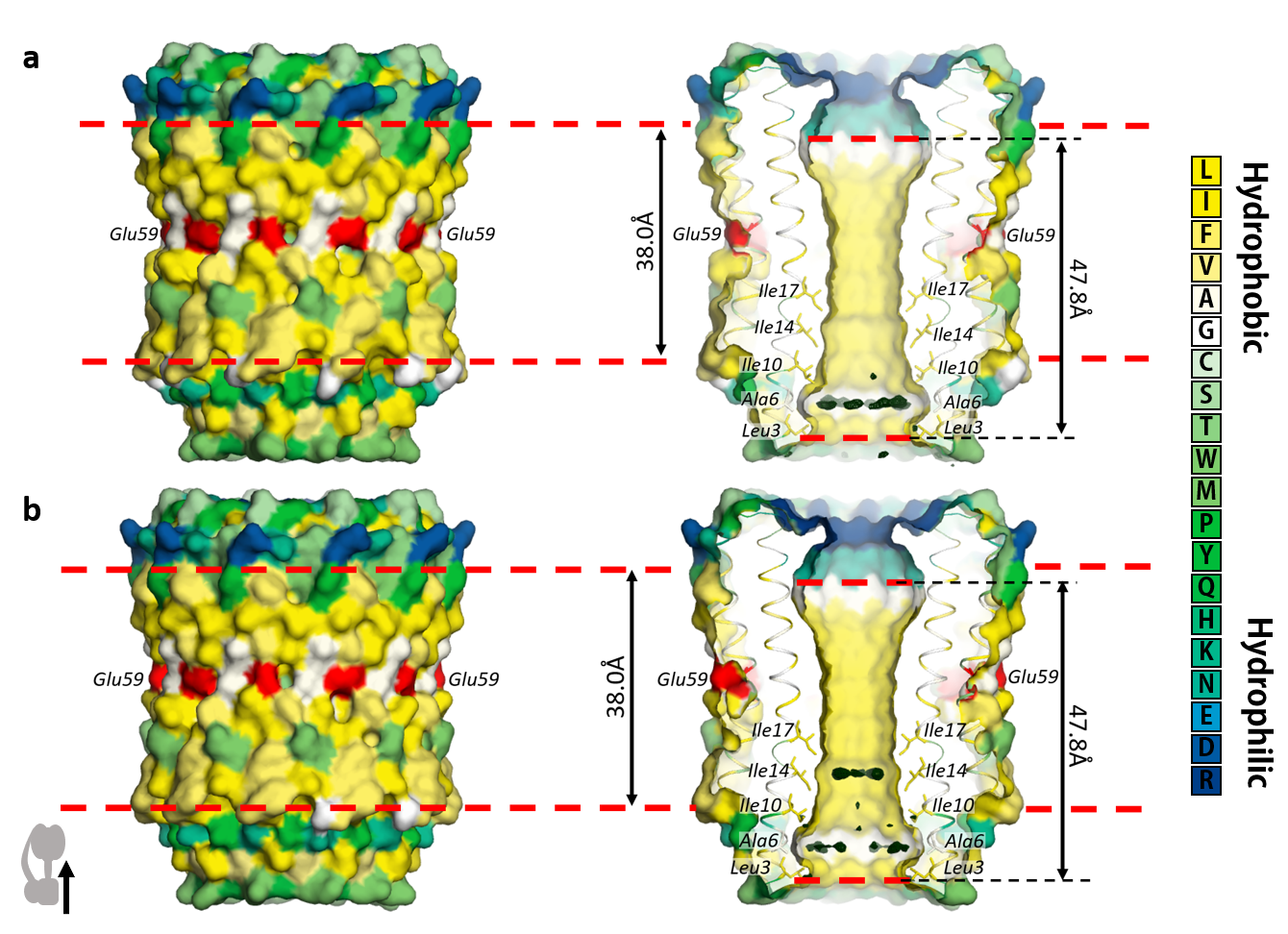
**

**Fig. S7.** Overall view of outer and inner surfaces of the yeast c-ring from mitochondria. **(A)** c_10_-ring from mitochondria of *Saccharomyces cerevisiae* at pH 5.5 (4F4S) and **(B)** at pH 7.5 (5BPS). Polar/apolar interfaces are marked by dashed red line. The membrane thickness is 38.0 and 47.8 Å for outer and inner surfaces, respectively. Additional electron densities are shown as deep green mesh, which represents the density map (Fo – Fc) at 3σ.

_
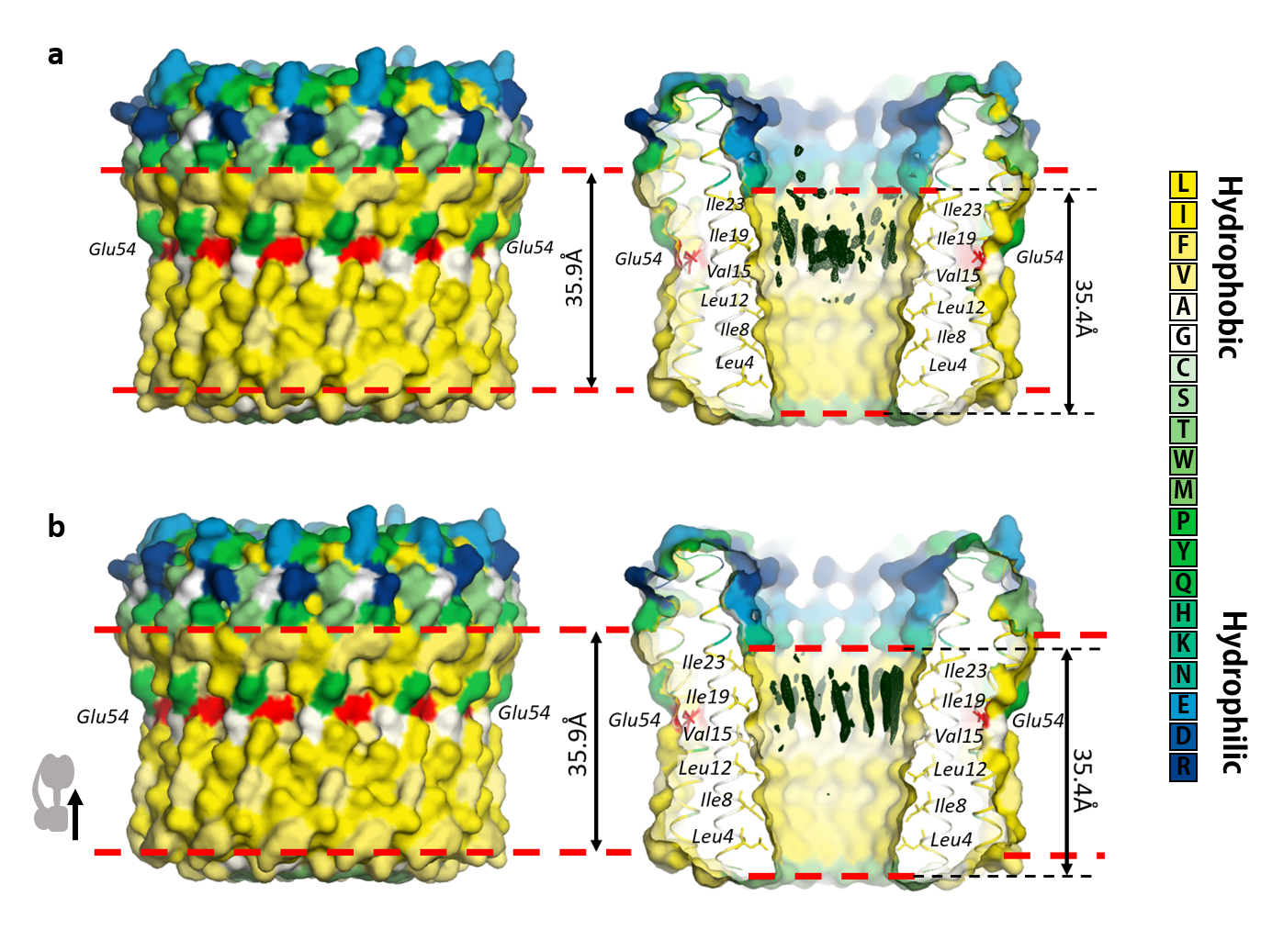
_

**Fig. S8.** Overall view of outer and inner surfaces of the bacterial c-ring. from *Bacillus pseudofirmus OF4.* **(A)** c_13_-ring from *Bacillus pseudofirmus OF4* at pH 4.3 (2X2V) and **(B)** at pH 9.0 (4CBK). Polar/apolar interfaces are marked by dashed red line. The membrane thickness is 35.9 and 35.4 Å for outer and inner surfaces, respectively. Additional electron densities are shown as deep green mesh, which represents the density map (Fo – Fc) at 3σ.


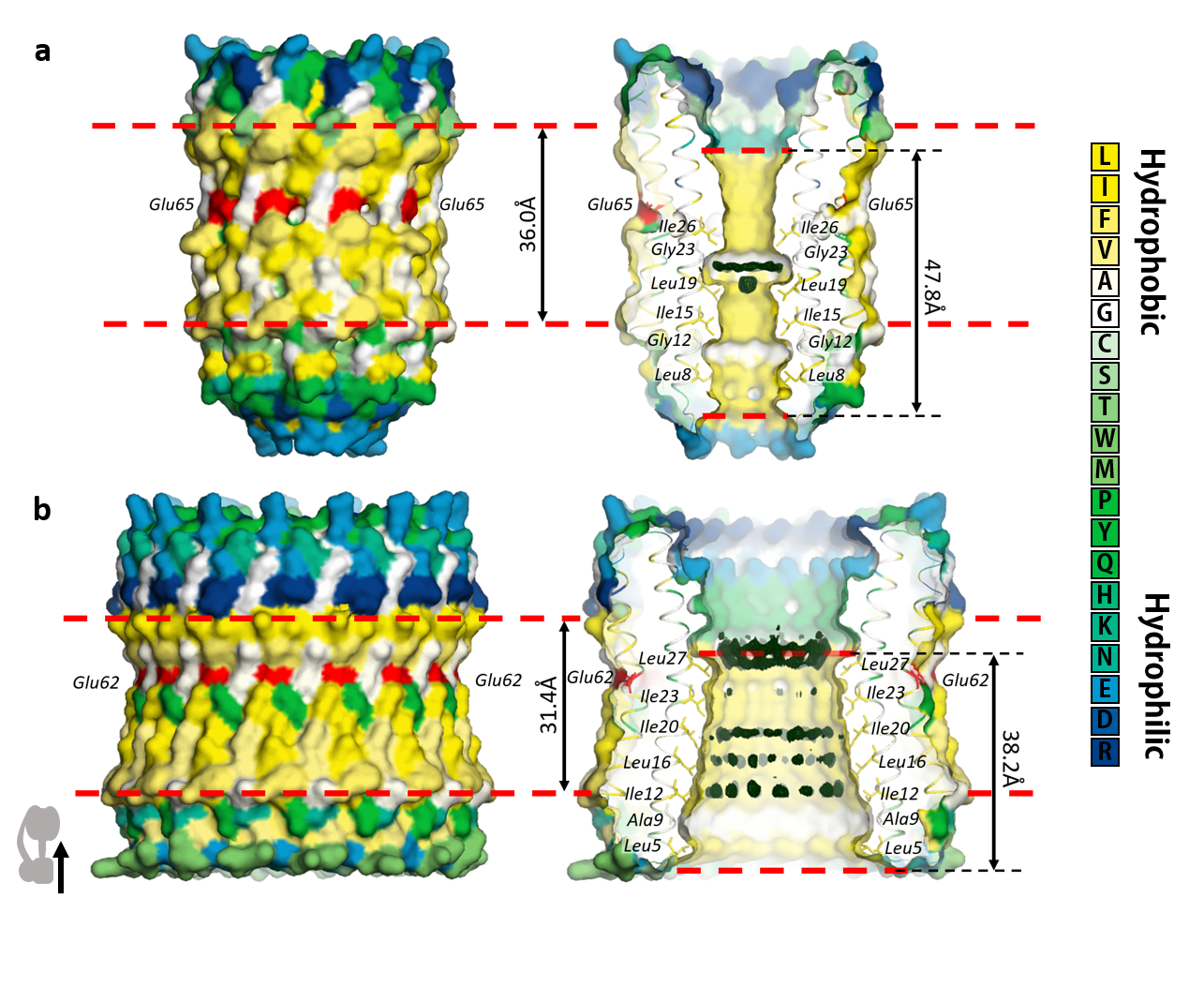


**Fig. S9.** Overall view of outer and inner surfaces of the other bacterial c-rings. **(A)** c_9_-ring from *Mycobacteria* at pH 8.0 (4V1H), the membrane thickness is 36.0 and 47.8 Å for outer and inner surfaces, respectively. **(B)** c_15_-ring from *Arthrospira platensis* at pH 4.3 (2XQU), the membrane thickness is 31.4 and 38.2 Å for outer and inner surfaces, respectively. Polar/apolar interfaces are marked by dashed red line. Additional electron densities are shown as deep green mesh, which represents the density map (Fo – Fc) at 3σ.

**
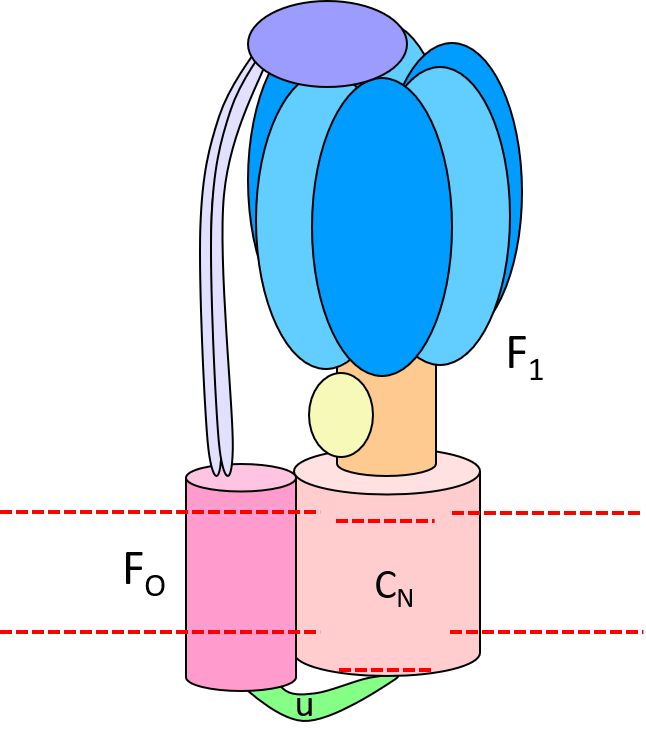
**

**Fig. S10.** New model of ATP synthase with unknown subunit (‘u’) connecting membrane F_O_ part with c-ring. Different subunits are colored with different colors. Putative polar/apolar interfaces are marked with red dashed lines. The length between polar/apolar interfaces is different inside and outside the c-ring. Unknown subunit ‘u’ connects membrane part of F_O_ with the c-ring stabilizing its rotation.


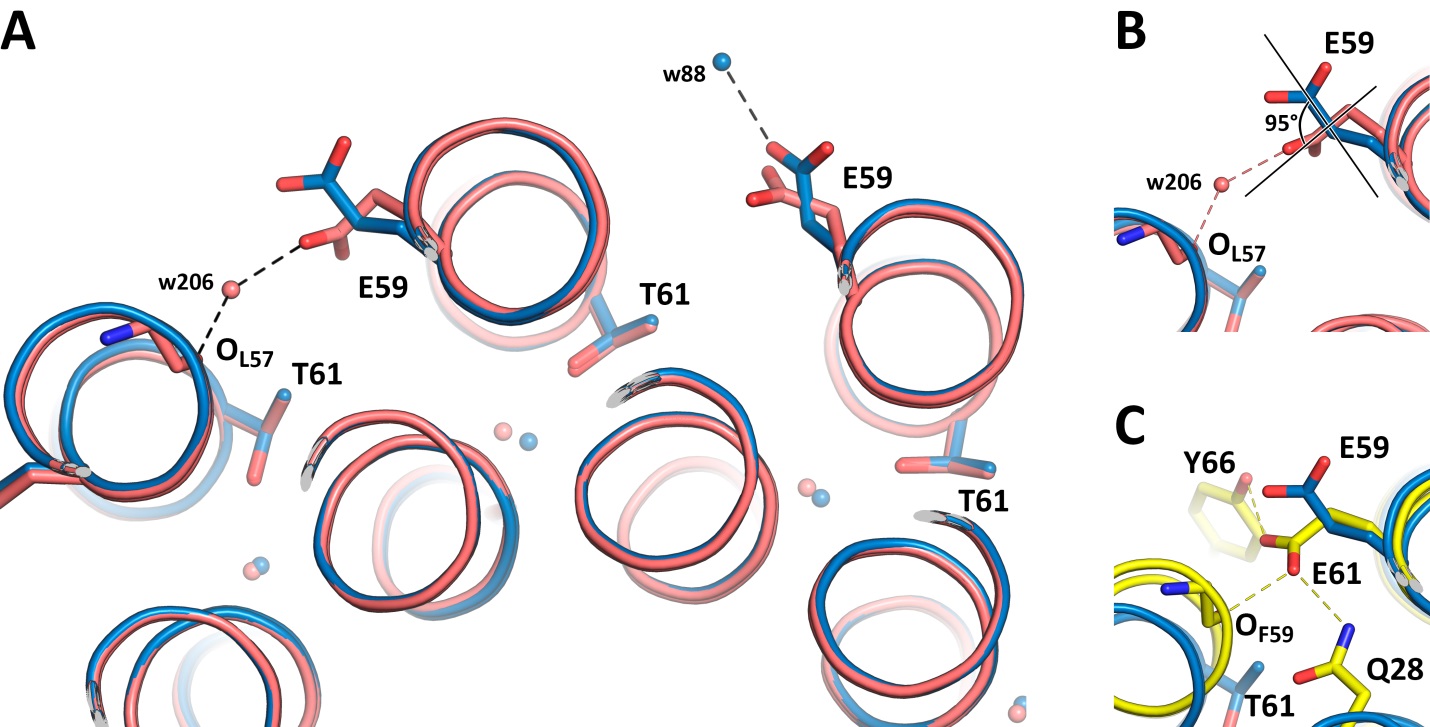


**Fig. S11.** “Opened” and “closed” states c-rings. **(A)** Structure alignment of “opened” (blue) and “closed” (red) states of yeast c_10_-ring (structures was taken from PDB: 3U2F – “opened”, 4F4S – “closed”). **(B)** Detailed view of the alignment of the active centers (Glu59) of yeast c_10_-ring. It is shown that the Glu residue changes the conformation, and the angle between CH_2_ – CH_2_ bonds is 95°. **(C)** Detailed view of the alignment of the active centers of “opened” state of yeast c_10_-ring and “closed” state of spinach c_14_-ring from this work (yellow) (Glu59 and Glu61, respectively). The view is from F_1_ part of ATP-synthase. Hydrogen bonds, stabilizing Glu residue in all structures are shown with dashed lines.


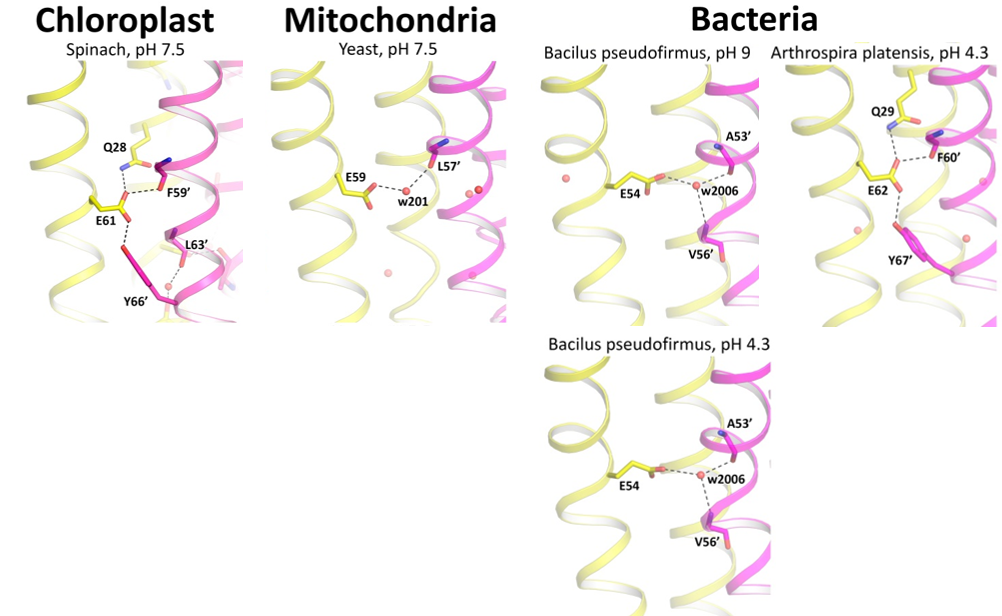


**Fig. S12.** Comparison of active centers of known H^+^ c-rings of high-resolution without ligands. The same orientation for each active center region is shown. Different *c* subunits are colored yellow and magenta respectively.


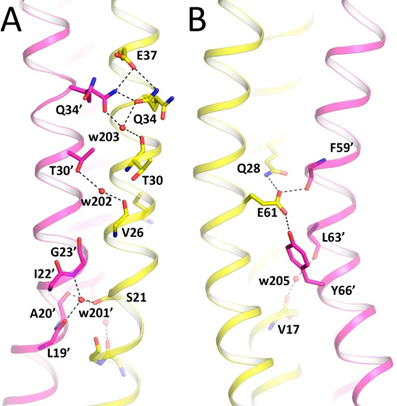


**Fig. S13.** Interactions between *c* subunits in the c-ring. **(A)** View from the inner pore. Two neighbor subunits are colored magenta and yellow. **(B)** View from the outside. Glu-61 is directly involved into intersubunit contacts.

**
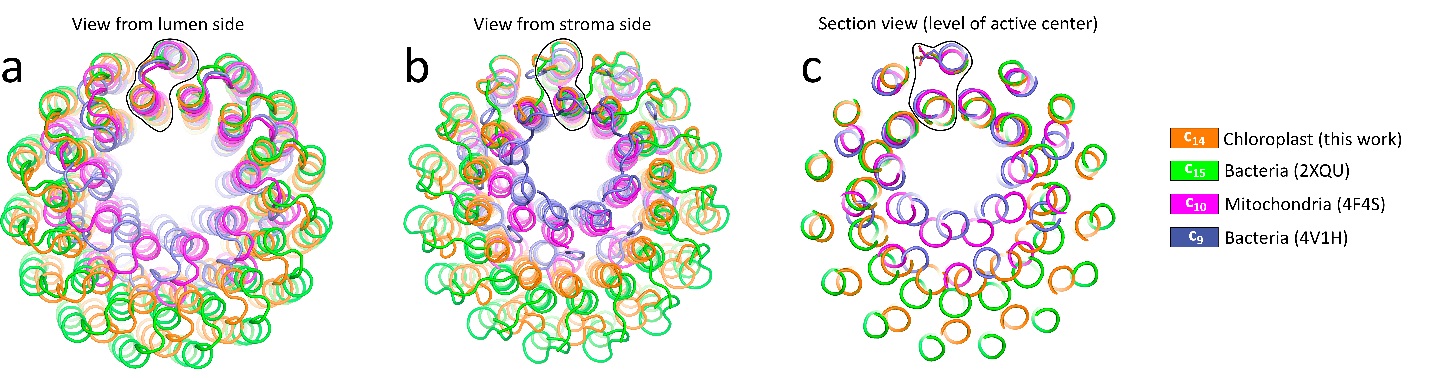
**

**Fig. S14.** Alignment of high-resolution c-ring structures from mitochondria, bacteria and chloroplast. **(A)** View from the lumen. **(B)** View from the stroma. **(C)** Section view at the level of the active center. Glutamate side chains are shown with sticks.


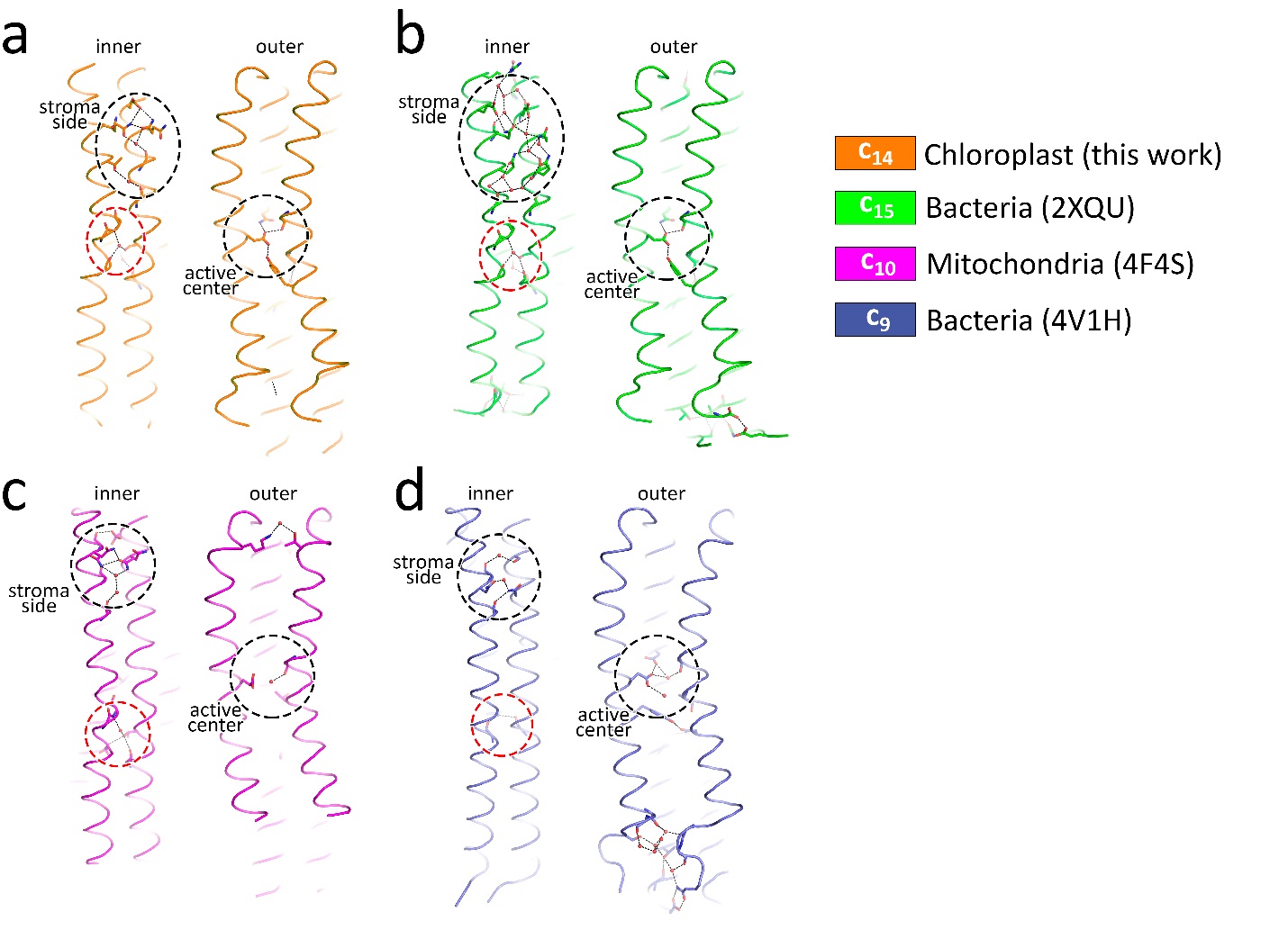


**Fig. S15.** Comparison of intersubunit contacts in different c-rings. **(A)** Chloroplast (this work). **(B)** Bacterial (PDB: 2XQU). **(C)** Mitochondrial (PDB: 4F4S). **(D)** Bacterial (PDB: 4V1H). The common contact regions are shown with dashed ellipses. The region at the level of active center inside the C ring is shown in red dashed ellipse.

**Table S2.** Comparison of different c-rings inner electron densities of all known H^+^ F-type ATP-synthases c-rings of high-resolution (better than 3.0Å). The *c* rings are divided by origin: bacterial, mitochondrial and chloroplast and shown as cartoon. Additional densities are shown as deep green mesh, which represents the density map (Fo – Fc) at 3σ. PDB ID, origin, number of protomer subunits and resolution are shown on the top and space group symmetry, pH of crystallization and authors are shown on the bottom of each structure.

**
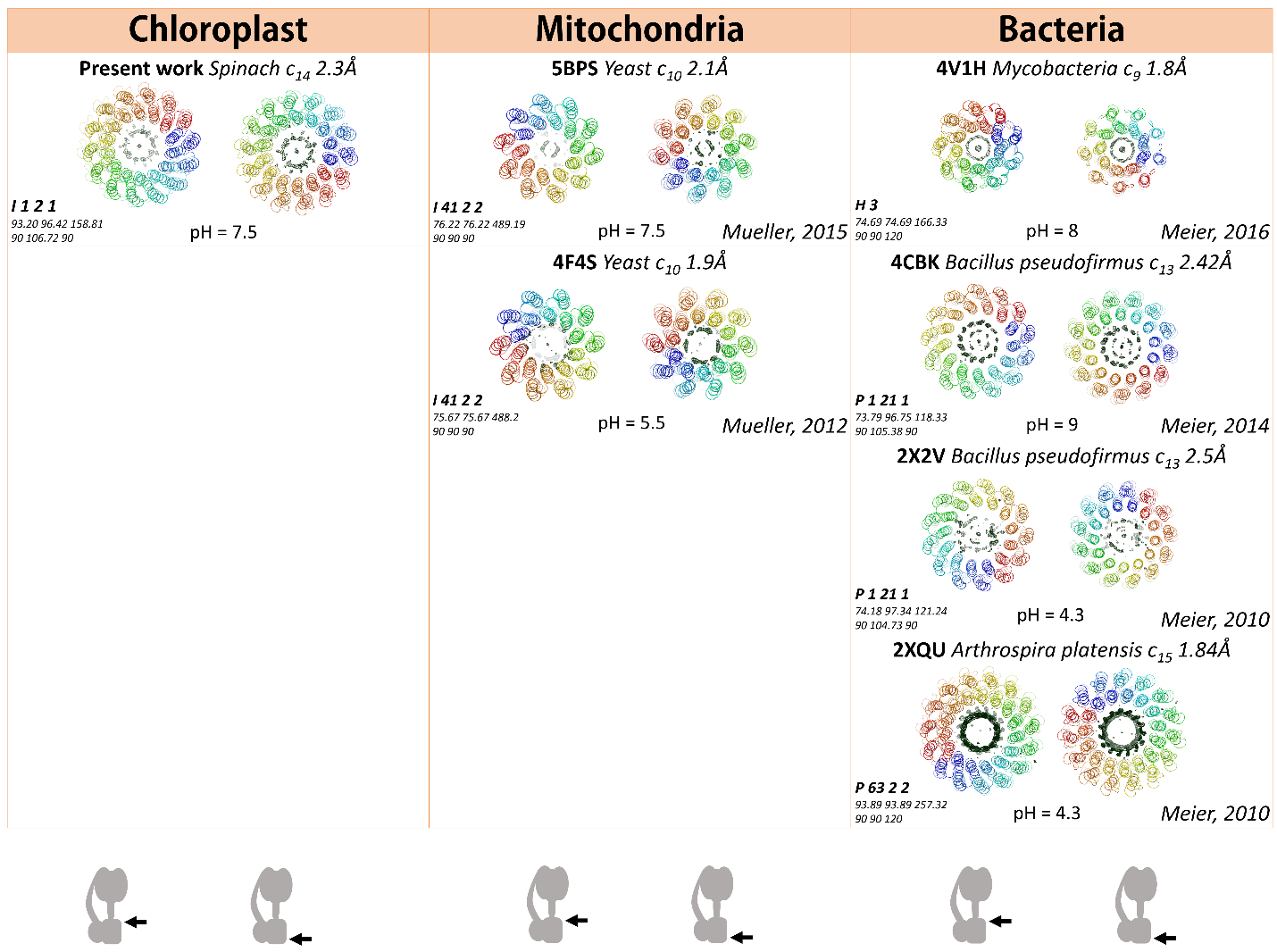
**

**Table S3.** Comparison of different c-rings purification and crystallization parameters. Chloroplast, mitochondria and bacteria c-rings are considered. Purification and crystallization conditions of the c-rings are listed.

| **№** | **PDB ID** | **Crystallization conditions** | **Sources** |
| --- | --- | --- | --- |
| ***1*** | Present work | Intact ATP-synthase was used for c-ring crystallization. Intact cF_1_F_O_ conc = 27.4 mg/mL; 0.1M MES pH 5.8, 1,4 M Ammonium Sulfate from the QCPI (Qiagen, Germany); 295K | *chloroplast* |
| ***2*** | 5BPS | Intact ATP-synthase was used for c-ring crystallization. Vapor Diffusion Sitting Drop; pH 7.5; 68% MPD, 8% propylene glycol, 0.3 M sodium chloride, 2 mM magnesium sulfate, 50 mM MES, pH 5.5; 298K | *mitochondria* |
| ***3*** | 4F4S | Intact ATP-synthase was used for c-ring crystallization. Vapor Diffusion Sitting Drop; pH 5.5; 68% MPD, 8% propylene glycol, 0.3 M sodium chloride, 2 mM magnesium sulfate, 50 mM MES, pH 5.5; 298K |  |
| ***4*** | 4V1H | C-ring was purified with harsh conditions (1.5%NLS, 60°C ). Vapor diffusion (hanging drops). Before setting up the vapor diffusion hanging drops, the protein was diluted 1:1 with 20 mM tris-HCl (pH 8.0) and 4% (w/v) OG. For cocrystallization, 0.35 mM BDQ was added to the protein solution. One microliter of the protein solution was then mixed with 0.5 ml of 28 to 30% PEG600 (polyethylene glycol, molecular weight 600). | *bacteria* |
| ***5*** | 4CBK | C-ring was purified with harsh conditions (1%NLS, 45°C). Hanging drops vapor diffusion method. 0.5 μl WT c13 ring (2.5 mg/ml) was supplied with 0.5% n-tridecyl-β-D-maltopyranoside and mixed with 0.25 μl crystallization mixture containing 0.1 M Tris/HCl pH 9.0, 20% (v/v) PEG 400. |  |
| ***6*** | 2X2V | C-ring was purified with harsh conditions (1%NLS, 65°C). Vapor diffusion in hanging drops at 18uC to a size of approx. 200x100x100 mm^3. The c-ring sample was supplied with 1% (w/v) of β-undecyl maltoside and mixed with crystallization buffer (0.1 M sodium acetate, pH 4.3) and 20% PEG 400 (v/v). Before flash-freezing in liquid nitrogen, the rod shaped clear crystals were transferred for 2 min into a buffer containing 30% PEG 400 (v/v), 0.1 M sodium acetate pH 4.5, and 0.05% b-dodecyl maltoside (w/v). |  |
| ***7*** | 2XQU | C-ring was purified with harsh conditions (1%NLS, 65°C). The purified c-ring sample with a protein concentration of 1 mg/ml was mixed with digalactosyl diacylglycerol (DGDG) from wheat (Larodan Fine Chemicals, Sweden) at a lipid-to-protein ratio of 0.4 (w/w) to yield a b-octyl-glucoside concentration of 0.6%. Bipyramidal crystals with dimensions 100 × 50 × 50 µm3 grew within 3–5 d by vapor diffusion in hanging drops, upon mixing the protein sample (1.8 mg/ml) in 2.5% (w/v) CYMAL-4–containing solution (in water) with crystallization buffer (10% (v/v) PEG 600, 350 mM Li2SO4, in 0.1 M sodium acetate buffer, pH 4.3). Before flash-freezing in liquid nitrogen, we transferred the crystals for 1 min into the same buffer but containing 35% (v/v) PEG 600. |  |

**Table S4.** Comparison of different c-rings parameters. Three classes of the c-rings (chloroplast, mitochondria and bacteria) are considered, **‘**C_N_’ corresponds the number of subunits in the c-ring, ‘Angle C_1_’ shows the angle that is formed by one c-subunit, D_in_ and D_out_ – inner and outer diameters of the c-ring respectively, P_in_ – the perimeter of the inner cavity and S_in_ – its area, Outer and Inner pol/apol distances show the distance between polar/apolar interfaces in outer and inner part of the c-ring respectively. N_C-C chains_ is the number of carbon chains that could fit the inner area S_in_ considering the area of one chain equals 20 Å^2^.

| **№** | **PDB ID** | **C_N_** | **Angle C_1_ [°]** | **D_in_ [Å]** | **D_out_ [Å]** | **P_in_ [Å]** | **S_in_ [Å^2^]** | **Outer pol/apol distance [Å]** | **Inner pol/apol distance [Å]** | **N_C-С chains_** | **Sources** |
| --- | --- | --- | --- | --- | --- | --- | --- | --- | --- | --- | --- |
| ***1*** | Our c-ring | 14 | 25.7 | 20.1 | 67.9 | 62.7 | 307.76 | 32.6 ± 0.8 | 45.8 ± 0.3 | 15.4 | *chloroplast* |
| ***2*** | 5BPS | 10 | 36.0 | 10.5 | 57.1 | 32.4 | 80.66 | 38.0 ± 0.8 | 47.8 ± 0.3 | 4.0 | *mitochondria* |
| ***3*** | 4F4S | 10 | 36.0 | 10.5 | 57.3 | 32.3 | 80.26 | 38.0 ± 0.8 | 47.8 ± 0.3 | 4.0 |  |
| ***4*** | 4V1H | 9 | 40.0 | 8.7 | 53.6 | 26.9 | 55.33 | 36.0 ± 2.2 | 47.8 ± 0.7 | 2.8 | *bacteria* |
| ***5*** | 4CBK | 13 | 27.7 | 19.5 | 67.7 | 60.8 | 288.53 | 35.9 ± 1.1 | 35.4 ± 1.6 | 14.4 |  |
| ***6*** | 2X2V | 13 | 27.7 | 19.8 | 68.1 | 61.5 | 294.82 | 35.9 ± 1.1 | 35.4 ± 1.6 | 14.7 |  |
| ***7*** | 2XQU | 15 | 24.0 | 21.8 | 72.0 | 68.1 | 363.54 | 31.4 ± 0.7 | 38.2 ± 1.5 | 18.2 |  |
